# SARS-CoV-2 infection reduces the number of spermatogonial stem cells and dysregulates the transcriptional landscape of the human testis

**DOI:** 10.21203/rs.3.rs-9371895/v1

**Published:** 2026-04-21

**Authors:** Miguel Brieno-Enriquez, Maria López-Panadés1, Ana Martinez-Marchal, Esther Choi, Andrew Levy, Tianjiao Chu, Shruthi Shivkumar, Justin Bochter, Juan Morales, Patrick Walsh, Marta Martin-Ruiz, Gretchen Rosado, Jimmaline Hardy, Yang Hu, Jiefei Wang, Cristina Madrid-Sandín, Andros Maldonado-Linares, Lidia Yang, Èlia Ramos-Ramells, Lisa Barton, Eric Duval, Edana Stroberg, Sanjay Mukhopadhyay, Uma R Chandran, Amanda Colvin Zielen, Kyle Orwig, Olivier Elemento, Carmen Marquez, Subha Ghosh, Lluis Bassas, Guilherme Costa, Ignasi Roig

**Affiliations:** University of Pittsburgh School of Medicine; Genome Integrity and Instability Research Group, Institut de Biotecnologia i Biomedicina (IBB), Universitat Autònoma de Barcelona, Spain; Magee-Womens Research Institute, Department of Obstetrics, Gynecology and Reproductive Sciences, University of Pittsburgh, Pittsburgh, USA; Magee-Womens Research Institute, Department of Obstetrics, Gynecology and Reproductive Sciences, University of Pittsburgh, Pittsburgh, USA; Magee-Womens Research Institute, Department of Obstetrics, Gynecology and Reproductive Sciences, University of Pittsburgh, Pittsburgh, USA; Magee-Womens Research Institute; Magee-Womens Research Institute, Department of Obstetrics, Gynecology and Reproductive Sciences, University of Pittsburgh, Pittsburgh, USA; Magee-Womens Research Institute, Department of Obstetrics, Gynecology and Reproductive Sciences, University of Pittsburgh, Pittsburgh, USA.; Magee-Womens Research Institute, Department of Obstetrics, Gynecology and Reproductive Sciences, University of Pittsburgh, Pittsburgh, USA.; Magee-Womens Research Institute, Department of Obstetrics, Gynecology and Reproductive Sciences, University of Pittsburgh, Pittsburgh, USA.; Magee-Womens Research Institute, Department of Obstetrics, Gynecology and Reproductive Sciences, University of Pittsburgh, Pittsburgh, USA.; Magee-Womens Research Institute, Department of Obstetrics, Gynecology and Reproductive Sciences, University of Pittsburgh, Pittsburgh, USA.; Magee-Womens Research Institute, Department of Obstetrics, Gynecology and Reproductive Sciences, University of Pittsburgh, Pittsburgh, USA.; Weill Cornell Medicine; University of Pittsburgh; Genome Integrity and Instability Research Group, Institut de Biotecnologia i Biomedicina (IBB), Universitat Autònoma de Barcelona, Spain.; Universitat Autònoma de Barcelona; Genome Integrity and Instability Research Group, Institut de Biotecnologia i Biomedicina (IBB), Universitat Autònoma de Barcelona, Spain.; Genome Integrity and Instability Research Group, Institut de Biotecnologia i Biomedicina (IBB), Universitat Autònoma de Barcelona, Spain.; Office of the Chief Medical Examiner, Oklahoma City, USA; Office of the Chief Medical Examiner, Oklahoma City, USA; Office of the Chief Medical Examiner, Oklahoma City, USA; Office of the Chief Medical Examiner, Oklahoma City, USA; University of Pittsburgh School of Medicine; Magee-Womens Research Institute, Department of Obstetrics, Gynecology and Reproductive Sciences, University of Pittsburgh, Pittsburgh, USA.; University of Pittsburgh School of Medicine; Weill Cornell Medicine; Clinica Gravida, Barcelona, Spain; Department of Anatomical Pathology, Cleveland Clinic, Cleveland, USA; Andrology Department, Laboratory of Andrology and Sperm Bank, Fundació Puigvert, Barcelona, Spain.; Universidade Federal de Minas Gerais; Autonomous University of Barcelona

**Keywords:** COVID-19, Spermatogonial stem cell, Sertoli cell, human testis, snRNAseq, brain

## Abstract

SARS-CoV-2 coronavirus emerged in 2019, leading to the Coronavirus disease 2019 (COVID-19). Expression of viral entry factors such as ACE2 and TMPRSS2 is higher in the testis, particularly in Sertoli cells, Leydig cells, and spermatogonia. To understand COVID-19’s impact on testicular cell populations and gene expression, we analyzed testicular tissue samples from 28 COVID-19 patients and compared them with 23 non-diseased controls. COVID-19 samples showed increased immune cell infiltration, thrombosis, and reduced numbers of testicular cells. There was a significant decrease in Sertoli cells and spermatogonial stem cells (SSC) among COVID-19 patients, associated with high levels of DNA damage and apoptosis in these cell types. To explore the pathways through which the virus affects testicular function, we profiled 112,657 single-nucleus transcriptomes from the testes of 4 COVID-19 patients and 4 controls. We found that COVID-19 infection alters multiple transcriptome clusters and induces a new COVID-19-specific cluster. To confirm that these transcriptome changes are COVID-19-specific, we compared our results with those from the brains of patients with COVID-19 and influenza. We observed an average of 144 dysregulated genes unique to COVID-19, regardless of tissue type. Lastly, we examined whether the SSC phenotype seen in fatal COVID-19 cases was also present in recovered patients. Indeed, recovered patients also exhibited high DNA damage and a reduced SSC population at 3, 6, and 12 months post-infection. Additionally, embryos derived from recovered patients’ sperm showed lower fertilization rates compared to control-derived embryos and fewer live births. Overall, our findings demonstrate that SARS-CoV-2 disrupts spermatogenesis, alters the transcriptional landscape, and affects human testicular architecture and function well after the acute phase of infection, with potential long-term consequences for male fertility.

## Introduction

Before 2003, human coronaviruses (HCoVs) were mainly associated with mild respiratory tract infections. However, the emergence of Severe Acute Respiratory Syndrome Coronavirus (SARS-CoV) in 2003 and Middle East Respiratory Syndrome Coronavirus (MERS-CoV) in 2012 revealed that coronaviruses can also cause severe and life-threatening infection^1^. In late 2019, a new coronavirus, known as SARS-CoV-2, emerged, causing the Coronavirus Disease 2019 (COVID-19), which spread worldwide, with more than 778 million confirmed cases and over 7 million deaths (as of 10^th^ December 2025). COVID-19 is mainly reported to cause a respiratory tract infection, but other organs, including the heart, liver, kidneys, gastrointestinal tract, brain, and skin, are also affected^2–10^. This heterogeneity may be explained by the expression of angiotensin-converting enzyme 2 (ACE2) in tissues, with organs that express higher levels of ACE2 being at greater risk of infection^11–14^. ACE2 functions as a regulator of the renin-angiotensin system (RAS), catalyzing the hydrolysis of angiotensin II into angiotensin-(1–7), which acts as a vasodilator and plays an essential role in modulating the cardiovascular system^15–17^. SARS-CoV-2 entry into the cells depends on its spike (S) protein binding to the ACE2 of the host cell, and on its subsequent priming by host proteases TMPRSS2 (transmembrane serine protease 2) and furin to promote virus-cell fusion and the subsequent release of viral RNA^1,18–22^. Internalization of surface ACE2 with the virus would increase circulating angiotensin II and consequent multiorgan damage^23,24^.

Interestingly, there is a sex-related bias in the disease, with men being more susceptible to a higher severity and mortality due to COVID-19 infection than women^25–27^. Plasma ACE2 levels are higher in men than in women, potentially making this population more vulnerable to SARS-CoV-2 infection. Similarly, TMPRSS2 expression is androgen-sensitive, being a mainly testosterone-regulated gene, possibly increasing the susceptibility to the disease in men^28–31^. Furthermore, men have higher basal expression of COVID-19 entry factors in their gonads, with the testes exhibiting higher ACE2 expression in the body^13,14^. Specifically, Sertoli cells, Leydig cells, and spermatogonia stem cells (SSCs) would have the highest expression of ACE2, TMPRSS2, and other factors in the testes^12,32,33^, potentially being highly permissive to viral entry and acting as a possible reservoir. Testes are an immune-privileged organ^34^. However, systemic viral infections can disrupt their function. Previous studies have shown the detrimental effects of Zika, Mumps, and immunodeficiency virus^35–38^ infections in the male genital system. In fact, SARS-CoV has been shown to cause orchitis, which damages the testicular structure and function^39,40^. Orchitis and hormonal changes have also been reported following SARS-CoV-2 infection^41,42 43,44^. Semen analyses of COVID-19 patients showed low sperm concentration^44–47^, low motility^43,44,47^, altered morphology^43,44^, higher sperm DNA fragmentation^43,48^, an increase in seminal leucocytes, and the presence of seminal inflammatory markers IL-6, IL-8, TNF-α, and MCP-1^45,46,49,50^. Histological analysis of testes from COVID-19-deceased patients revealed fibrosis and edema in the interstitial tissue with infiltration of CD3+ T lymphocytes and CD68+ macrophages; sloughing of the cells into the lumen of the tubule; swelling and vacuolation of Sertoli cells; loss of germ cells and an increase in apoptosis inside the tubules^45,50–52^.

The deleterious effects of viruses on SSCs and Sertoli cells could severely impact fertility^53,54^. SSCs are the most undifferentiated spermatogonia, carrying two vital functions: self-renewal of the population and differentiation into daughter spermatogonia, which will eventually further differentiate, enter meiosis, and produce sperm^55,56^. In this sense, reduction of the SCC pool, defects in their cell division, or differentiation will lead to subfertility or infertility. Sertoli cells participate in and support almost every aspect of spermatogenesis, directly determining the number of SSCs and, hence, the number of germ cells^57–59^. We hypothesize that SARS-CoV-2 infection disrupts the testicular structure, changing the transcriptional landscape and reducing the population of cell types with higher ACE2 expression, namely SSCs and Sertoli cells.

Herein, we show that both in fatal samples with severe COVID-19 and in COVID-19 recovered patients, there is a reduction in SSCs and Sertoli cells and an activation of the DNA damage response, as shown by an increase in the γH2AX signal. Histological changes are also associated with disruption of the transcriptomic landscape in SARS-CoV-2-infected testes. Bioinformatic analyses also demonstrate that the virus’s detrimental effects are not solely due to systemic inflammation but are mainly driven by a specific COVID-19 viral signature. Finally, we also show the persistence of the reduction in SSC and Sertoli cells in testicular biopsies from COVID-19-recovered patients up to 12 months post-infection, and the effects of the fertility treatments these patients received on reproductive outcomes. These findings suggest that COVID-19 may induce long-lasting alterations in testicular architecture and function, potentially impacting male fertility well beyond the acute phase of infection.

## Results

### SARS-CoV-2 infection disrupts the testicular structure.

We analyzed the impact of acute COVID-19 infection on postmortem patient testes and compared them with non-infected controls. All the enrolled patients (aged from 25 to 88 years old) deceased from COVID-19 complications; SARS-CoV-2 infection was confirmed by SARS-CoV-2 RT-qPCR from oropharyngeal and nasopharyngeal swabs. Histological analyses were performed using samples from unvaccinated patients who died of COVID-19 in the U.S., Spain, and Brazil (n= 28; Supplementary Table 1). Samples of testes from males who died in the U.S. and Spain by causes such as car accidents, anoxia, cerebrovascular accidents, cardiac arrests, and most of which were collected before the onset of the COVID-19 pandemic, were used as controls (n= 23; Supplementary Table 1). We tested for the presence of the virus in the testes using a commercial quantitative real-time PCR (qRT-PCR) kit that targets two regions of the SARS-CoV-2 genome (N1 and N2). As previously published, we detected viral RNA in a portion of COVID-19 samples (21.4%; Supplementary Table 2).

**Figure 1:**
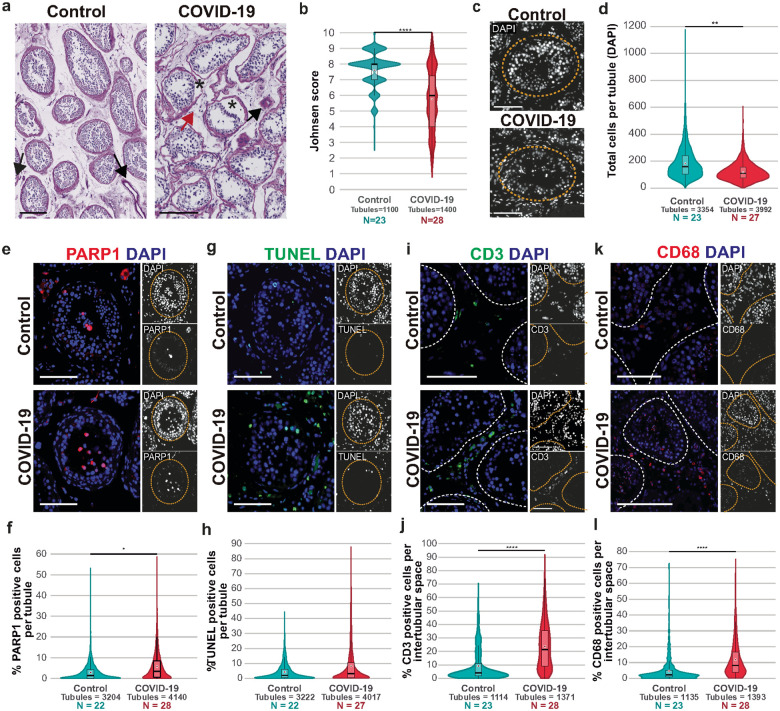
Legend not included with this version.

To evaluate the impact of COVID-19 on the testicular morphology, we used PAS-hematoxylin-stained testes sections. COVID-19 patients showed alterations of the seminiferous tubules, such as detachment of the basement membrane, with cell loss and sloughing into the lumen (Fig. 1a). To systematically evaluate the severity of the testicular damage caused by COVID-19, the tubules were classified using the Johnsen scoring system, which ranges from 10 to 1, being 10 a tubule with fully normal spermatogenesis and 1 a tubule with no seminiferous epithelium, with the correspondent intermediate scores (see materials and methods for more details)^60^. The Johnsen score showed a statistically significant decrease of germ cells in COVID-19 tubules, with a median score of 6, compared to the median score of controls of 8 (Fig. 1b, Supplementary Fig. 1a, Supplementary Table 3, p-value = 3.55E-06, Wilcoxon rank-sum test).

Using 4′,6-diamidino-2-phenylindole (DAPI) staining, which marks the cell nucleus, we counted the total number of cells per tubule in Control (N=3,354 tubules from 23 patients) and COVID-19 patients (N=3,992 tubules from 27 patients; the 28^th^ patient was excluded because it was an outlier). COVID-19 patients showed a reduction of 34% in the cell number (median = 110 cells per tubule) compared to control testes (median = 159.5 cells per tubule; Fig. 1c–d & Supplementary Fig. 1b, Supplementary Table 3, p-value = 0.0020, Welch’s test).

Next, we evaluated cell number reduction by performing immunofluorescence (IF) with the cell death marker Poly(ADP-ribose) polymerase 1 (PARP1). The PARP1-mediated cell death cascade is initiated by DNA damage, leading to loss of genomic integrity and culminating in cell death if the damage cannot be repaired^61^. COVID-19 patients had twice as many PARP1+ cells than controls. COVID-19 tubules had a median of 4 PARP1-positive cells per tubule compared to 2 cells per tubule in control samples (Fig. 1e–f & Supplementary Fig. 2a, Supplementary Table 3, p-value = 0.0120, Wilcoxon rank-sum test). To confirm these results, we checked for the presence of DNA fragmentation using TUNEL assay, as a readout of later stages of apoptosis^62^. COVID-19 patient testes had almost double of TUNEL-positive cells than controls (Fig. 1g–h & Supplementary Fig. 2b, Supplementary Table 3, p-value = 0.0629, Wilcoxon rank-sum test).

Intratubular defects in COVID-19 were associated with interstitial tissue lesions; samples from COVID-19 patients showed the presence of thrombi, enlarged blood vessels, red cell exudation, edema, and infiltration of white blood cells. To evaluate infiltration of interstitial tissue by white blood cells, we performed IF staining for the T-cell marker CD3 and the macrophage marker CD68. COVID-19 patients showed 160.4% more CD3+ cells than controls (COVID-19 median = 7 cells, Control median = 2 cells; Fig. 1i–j & Supplementary Figure 2c, Supplementary Table 3, p-value = 2.75E-05, Wilcoxon rank-sum test). Similarly, a rise of 156.5% was observed for CD68+ in COVID-19 patients (COVID-19 median = 3 cells; Control median = 1 cell; Fig. 1k–l & Supplementary Fig. 2c, Supplementary Table 3, p-value = 3.25E-05, Wilcoxon rank-sum test). Overall, these results indicate that COVID-19 induces a reduction of intratubular cells, apoptosis, and infiltration of white blood cells into the testicular interstitial space.

### Sertoli cells display higher levels of DNA damage and apoptosis in fatal COVID-19 patients.

**Figure 2:**
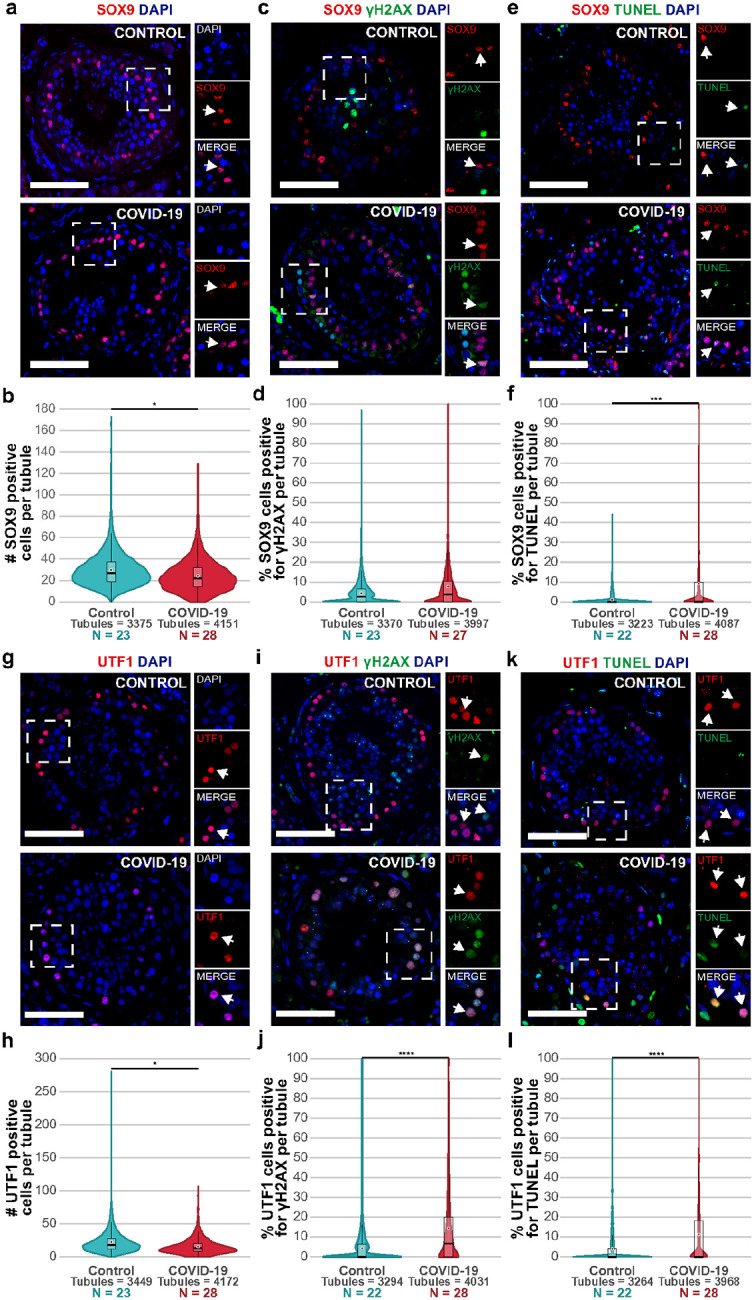
Legend not included with this version.

**Figure 3:**
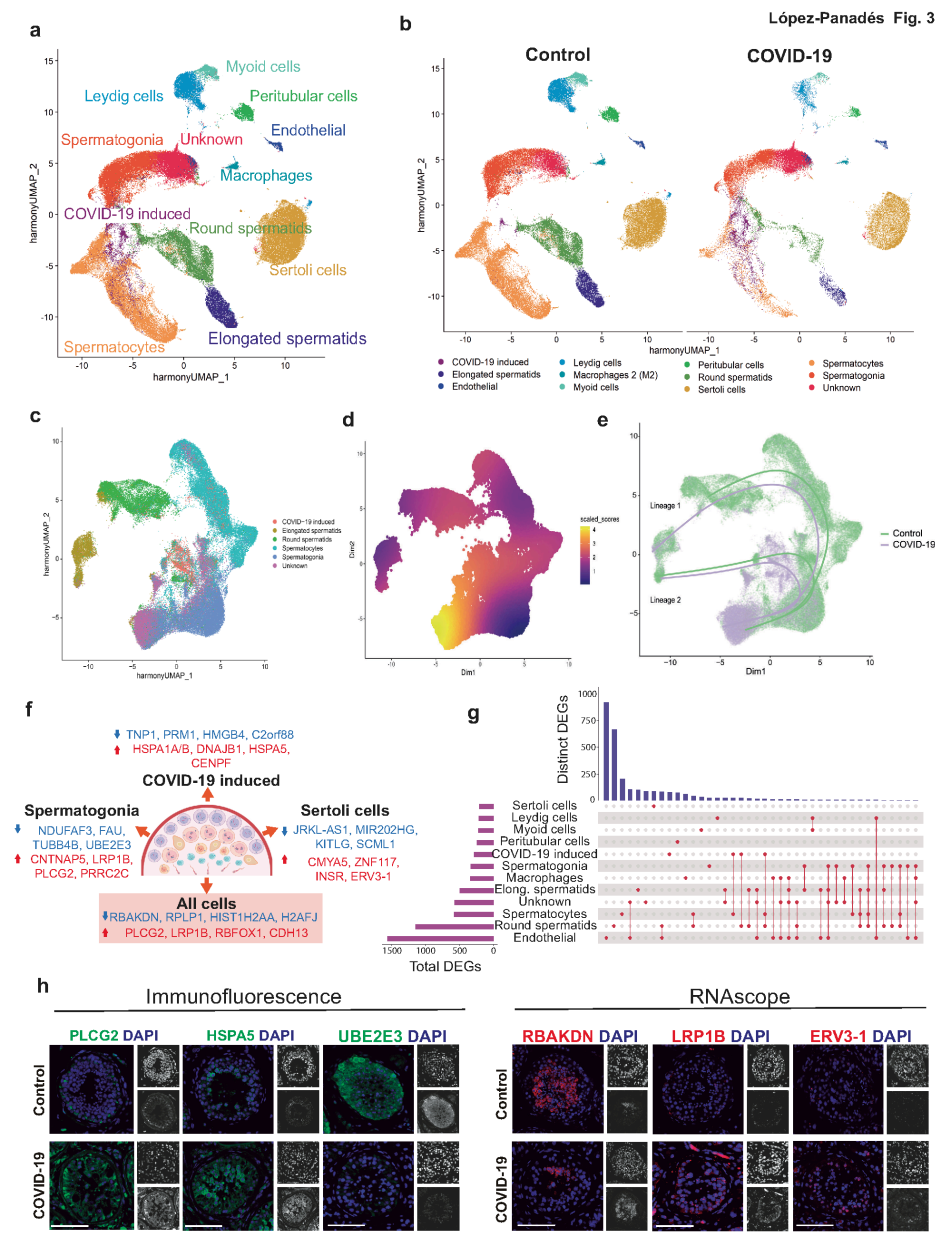
Legend not included with this version.

Sertoli cells are somatic support cells of the germinal epithelium of the testis that play an essential role in regulating, promoting, and advancing spermatogenesis and forming the blood-testis barrier^63,64^. Moreover, Sertoli cells highly express ACE2, making them a possible entry site for SARS-CoV-2^12,32^. Previous studies showed that COVID-19 causes swelling, vacuolation, and cytoplasmic rarefaction of Sertoli cells, but without altering their numbers^49,50^. To assess the impact of SARS-CoV-2 infection on Sertoli cells, we first used PAS-hematoxylin-stained samples to analyze the general morphology of Sertoli cells. We observed frequent Sertoli cell vacuolation (Fig. 1a). Next, we used IF against the Sertoli cell marker SRY-box transcription factor 9 (SOX9) to quantify the total number of cells per tubule. We observed a significant decrease in the number of SOX9+ cells in COVID-19 samples compared to controls (COVID-19 mean = 24.22 cells; Control mean = 29.77 cells; Fig. 2a–b & Supplementary Fig. 3a, Supplementary Table 3, p-value = 0.0211, Student’s t-test). To explore if COVID-19 induces DNA damage in the Sertoli cells, we performed a co-staining against SOX9 and phospho-histone H2AX (Ser139) (gH2AX) to detect double-strand breaks^65^. 4.34% of SOX9+ cells were also positive for gH2AX in tubules from control patients, while 8.36% of SOX9+ cells were double positive in COVID-19 samples (Fig. 2c–d & Supplementary. Fig. 3b, Supplementary Table 3, p-value = 0.0616, Wilcoxon rank-sum test), suggesting that SARS-CoV-2 infection likely induces DNA damage in Sertoli cells.

Next, we evaluated if the detection of SOX9+gH2AX+ cells was associated with higher apoptosis rates, which could explain the decrease in Sertoli cells in COVID-19 patients. Both Control and COVID-19 samples showed a few tubules with cells positive for both SOX9 and TUNEL However, COVID-19 samples presented 346.42% more double-positive cells than controls (COVID-19 mean = 1.25 cells per tubule; Control mean = 0.28 cells per tubule; Fig. 2e–f & Supplementary Fig. 3c, Supplementary Table 3, p-value = 0.0005, Wilcoxon rank-sum test), suggesting that COVID-19 induced apoptosis of Sertoli cells.

### COVID-19 infection induces DNA damage and apoptosis in SSCs.

SSCs are the germ cell stem cells in the testis, able to self-renew and generate committed spermatogonia to differentiate into spermatocytes, initiate meiosis, and finally generate sperm^55,56^. Defects in SSC cell division or differentiation are directly associated with subfertility and infertility^66,67^. Previous reports showed that SSCs exhibit high expression of ACE2 and TMPRSS2, making them a potential entry site for SARS-CoV-2^12,32^. To test the impact of SARS-CoV-2 on SSCs, we performed IF against the SSC maker undifferentiated embryonic cell transcription factor 1 (UTF1). We observed a statistically significant reduction of 27.7% of UTF1+ cells in COVID-19 patients (median = 13 SSCs per tubule), compared to controls (median = 18 SSCs per tubule; Fig. 2g–h & Supplementary Fig. 4a, Supplementary Table 3, p-value = 0.0218 Wilcoxon rank-sum test), suggesting that COVID-19 reduces the amount of SSC in the seminiferous tubules.

Next, we addressed the cause of this reduction. First, we evaluated whether COVID-19 induced DNA double-strand breaks in SSCs by staining tissues with UTF1 and gH2AX. We observed that COVID-19 samples had 14.4% of the SSCs per tubule positive for gH2AX (median = 1 cell), while the control samples had 4.3% of positive SSCs per tubule (median = 0 cells; Fig. 2i–j & Supplementary Fig. 4b, Supplementary Table 3, p-value = 0.0002, Wilcoxon rank-sum test). Next, we evaluated if the increased DNA damage in the SSCs was associated with increased apoptosis. Thus, we marked apoptotic SSCs using TUNEL assay followed by UTF1 immunostaining. We found that 11.3% of the SSCs were double-positive for UTF1+TUNEL+ in COVID-19 (median = 0 cells), but only 4% of the control samples were UTF1+ TUNEL+ (median = 0 cells; Fig. 2k–l & Supplementary Fig. 4c, Supplementary Table 3, p-value = 3.57E-05, Wilcoxon rank-sum test).

### Disruption of the testicular transcriptional landscape in acute COVID-19 infection

To understand how the virus affects the transcriptional landscape in testes, we isolated the nuclei and performed a single nuclei RNA sequencing (snRNAseq) from five acute fatal COVID-19 patients and five control non-diseased testes autopsies. After quality control filtering (QC), we removed two samples (one control sample and one COVID-19 sample) due to their poor quality (high mitochondrial gene content and low gene numbers), finally obtaining a total of 112,657 nuclei (77,581 in control, 35,076 in COVID-19) with an average of 1,743.9 genes (2,320.7 in control, 1,167.2 in COVID-19) (Supplementary Fig. 5a). The samples were matched in age, with both groups between 45 and 65 years old (Supplementary Fig. 5b), and in RNA quality. The percentage of mitochondrial genes was higher in controls. Still, in both groups, the cutoff was set to be lower than 5% (Supplementary Fig. 5b). The unsupervised clustering yielded ten different clusters representing the various cell types in the testes with previously established markers^68–70^: macrophages, endothelial cells, peritubular cells, myoid cells, Leydig cells, Sertoli cells, spermatogonia, spermatocytes, round spermatids and elongated spermatids (Supplementary Table 4–5a). In addition to the standard clusters in the testis, we obtained two extra clusters. The first one containing 312 control cells and 2,636 COVID-19 cells. Hence, as 89.4% of the total cells come from the COVID-19 condition, we called this cluster “COVID-19 induced” cluster. We also obtained a second cluster with 5,470 control cells and 7,982 COVID-19 cells, comprised of an unknown cell type, sharing expression markers with endothelial cell and spermatogonia clusters (Unknown cluster) (Fig. 3a, Supplementary Fig. 5c-e). Separating the data by condition, we observed that the COVID-19 testes had a general loss of cells, affecting, specifically, the meiotic and post-meiotic cell populations (spermatocytes, round spermatids, and elongated spermatids, Fig. 3b, Supplementary Fig. 5c). These data confirm our previous finding showing that acute COVID-19 causes the loss of germ cells and also suggests that SARS-CoV-2 infection alters the global transcriptome of the testis.

### A second cell lineage is observed comprised mainly of cells from the two unique clusters

To infer whether there are any differences in the trajectory and cell fates of spermatogenic cells, we ran a trajectory inference with condiments^71^ with our snRNAseq data; specifically, with the Unknown, Spermatogonia, Spermatocytes, Round spermatids, Elongated spermatids and COVID-19 induced clusters (Fig. 3c). Firstly, we determined the differential topology between the control and COVID-19 cohorts. Highly imbalanced areas were detected between cohorts in the area comprising the Unkown cluster and the region that corresponded to the COVID-19 induced cluster (Fig. 3d).

Next, a topology test assessment was performed. The null hypothesis was rejected, thus implying that in slingshot, separate trajectories per each condition would be obtained. Indeed, two lineages with two trajectories each were obtained (Fig. 3e). Lineage 1 followed the expected progression of spermatogenic cells: from spermatogonia, to spermatocytes, to round spermatids, to finish with elongated spermatids. Interestingly, for the COVID-19 group, the lineage 1 started further back, into the Unknown cluster. The lineage 2 started from the Unknown cluster, it then went into spermatogonia and slightly into the spermatocyte cluster, and then into the COVID-19-induced cluster, and ended up at the elongated spermatid cluster without following the usual route of differentiation (Lineage 1).

Next, we wanted to know whether cells from both groups were equally represented along the pseudotime for a given lineage (Supplementary Fig. 6a), with the developmental stage of each cell being estimated using its pseudotime^72^. In both lineages, we could observe an effect that could be induced by the infection. For lineage 1, COVID-19 cells progressed more slowly and remained in the initial stages of differentiation, correlating with the Unknown and Spermatogonia clusters. On the other hand, the control group appeared to progress normally, with a low cell density at the beginning and a normal progression from spermatogonia to elongated spermatids. For lineage 2, there also seemed to be an imbalance between groups. The control group exhibited a low cell density at the beginning of the pseudotime, likely corresponding to the Unknown cluster, followed by a higher density corresponding to spermatogonia, spermatocytes, and elongated spermatids. The COVID-19 group, in contrast, showed a higher density at the start and halfway through the pseudotime, which could correlate with the COVID-19-induced cluster. Lastly, there were almost no cells in the region where elongated spermatids were located. This suggested that there was a global difference across all lineages, mainly driven by differences in distribution across lineage 2.

When examining the distribution of weights (Supplementary Fig. 6b), we found that most cells in the control group belong solely to lineage 1 (weight of 1.0), while some cells are assigned to both lineages (weight of 0.5). Because the weights for both lineages are symmetrical, we concluded that most control cells do not belong to lineage 2 (weight of 0.0). In contrast, the weight distribution in the COVID-19 group shows that most cells are assigned to both lineages, whereas fewer cells are exclusively assigned to either lineage 1 or 2. Based on these findings, we suggest that germ cells from COVID-19 patients progress through spermatogenesis less efficiently, leading to a relative accumulation of germ cells at the spermatogonial phase, with an unknown cell population being delayed longer before the onset of spermatogenesis. Surprisingly, a second lineage is observed, mainly comprising cells from the COVID-19 group, extending from the unknown cluster to elongated spermatids, passing through spermatogonia and COVID-19-induced clusters. These data suggest the unknown cluster is transcriptionally similar to the spermatogonia cluster. At the same time, the COVID-19-induced cluster might have a similar enough transcriptional profile to connect spermatogonia and elongated spermatids, suggesting this cluster could consist of spermatocytes and/or round spermatids that exhibit altered transcriptional landscapes upon COVID-19 infection.

### Changes in the differentially expressed genes after SARS-CoV-2 infection.

Next, we identified the differentially expressed genes (DEGs) in COVID-19 samples. An initial analysis pooling all cells together showed 290 DEGs, 190 downregulated and 100 upregulated (Supplementary Fig. 7a). We validated these results by confirming the expression of a selection of the top downregulated genes such as RBAK Downstream Neighbor (RBAKDN), Ribosomal Protein Lateral Stalk Subunit P1 (RPLP1), the histone HIST1H2AA and H2AFJ; and a selection of the top upregulated genes like Phosphatidylinositol-specific phospholipase C gamma 2 (PLCG2), the LDL Receptor Related Protein 1B (LRP1B), the RNA Binding Fox-1 Homolog 1 (RBFOX1) and Cadherin 13 (CDH13) by RNAscope and IF (Fig. 3f–h, Supplementary Fig. 7b, Supplementary table 6).

**Figure 4:**
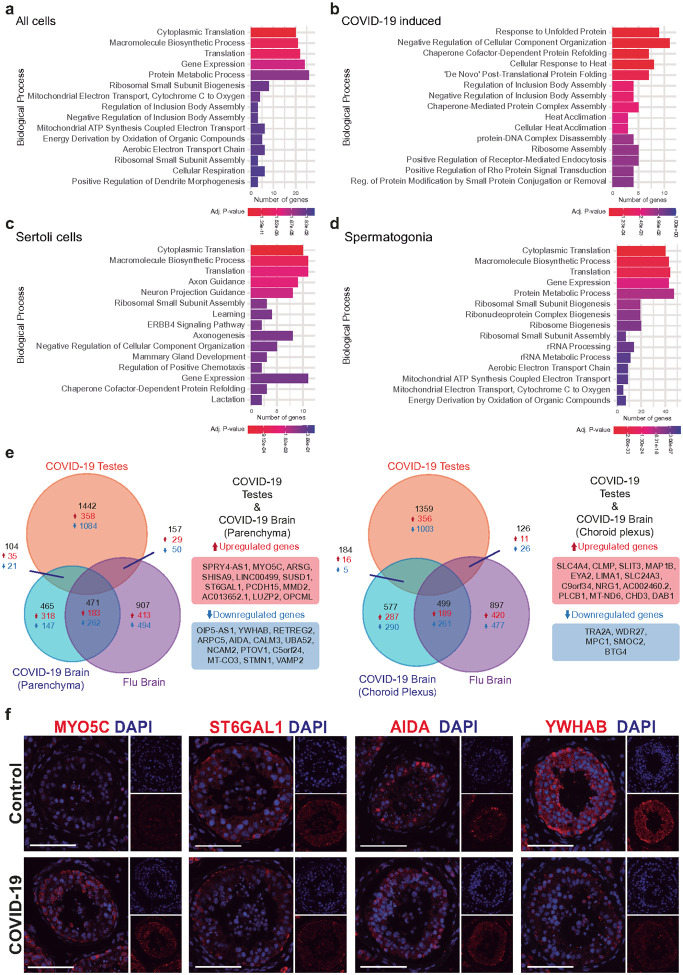
Legend not included with this version.

Gene Ontology (GO) analysis of all DEGs across all testicular cell types revealed that the top 15 most significantly enriched biological processes were primarily associated with protein synthesis and gene expression (including cytoplasmic translation, macromolecule biosynthetic process, translation, gene expression, and protein metabolic process), which are related to virion particle synthesis (Fig. 4a, Supplementary Table 5c). Consistently, terms related to the biogenesis and assembly of ribosomal subunits were also enriched. Most of the GO top hits were also hallmarks of viral infection, such as the rewiring of mitochondrial respiration and ATP synthesis (e.g., energy acquisition, the electron transport chain, and cellular respiration), or the formation of replication factories for viral production (e.g., regulation of inclusion body assembly).

When analyzing DEGs by expression direction, downregulated DEGs were strongly enriched in processes related to cytoplasmic translation, macromolecule biosynthesis, translation, gene expression, and energy obtention through cellular respiration (Supplementary Fig. 8a, Supplementary Table 5c), suggesting a global suppression of protein synthesis machinery. These GO terms map onto known SARS-CoV-2 strategies. Studies in other tissues have shown that the virus suppresses host ribosome biogenesis and translation and rewires mitochondrial respiration^73,74^. Moreover, DEGs related to the binding of sperm to zona pellucida were also downregulated, suggesting that SARS-CoV-2 infection could also alter the spermatogenesis-specific transcriptional program (Supplementary Fig. 8a, Supplementary Table 5c).

In contrast, the upregulated DEGs in COVID-19 patients were associated with more diverse processes, including regulation and negative regulation of inclusion body assembly, heat acclimation and cellular response to heat, protein refolding through chaperones and *de novo* post-translational protein folding, positive regulation of TNF-mediated signaling pathway and regulation of Nucleotide-Binding Oligomerization Domain Containing 2 (NOD2) signaling (Supplementary Fig. 8a, Supplementary Table 5c). These GO terms indicate a stressed, inflamed testis mounting a chaperone/heat-shock- and innate immune-response.

Next, we performed cluster analysis after analyzing the global DEGs (Supplementary Table 5b). We observed that the most dysregulated genes were from Endothelial cells, with 404 downregulated genes, and 1,157 upregulated genes (Supplementary Fig. 7a, Supplementary Table 5b). The meiotic and post-meiotic clusters (spermatocytes, round spermatids, and elongated spermatids) showed a high number of downregulated genes (401, 560, and 335, respectively), which correlates with the decrease in the number of cells in these clusters (Supplementary Table 5b). To visualize the intersections of the data sets, we plotted the data into an upset plot (Figure 3g). Interestingly, the endothelial cell cluster had the highest number of DEGs, with more shared connections to other clusters, both somatic and meiotic, involving 12 shared nodes. Conversely, the Sertoli cell cluster had the fewest total DEGs (105 downregulated and 100 upregulated) (Supplementary Table 5b) and no connections to other clusters, suggesting that the up- and down-regulated genes were highly specific for Sertoli cells. We also observed that the COVID-19-induced cluster shared DEGs with the Spermatocytes and Round spermatids clusters. This data suggests that this cluster may comprise meiotic and early post-meiotic cells that the SARS-CoV-2 infection has transcriptionally modified.

### The COVID-19-induced cluster presents distinctive DEGs.

As mentioned above, almost 90% of the total cells from the COVID-19-induced cluster are from COVID-19 patients. Thus, we wanted to learn more about this cluster and analyze its transcriptome. First, we confirmed the snRNAseq findings by analyzing the expression of C2orf88, HSPA5, DNAJB1, and STIP1 by IF. In all cases, we observed a consistent trend in protein expression, as indicated by the sequencing results (Fig. 3h, Supplementary Figure 7b).

Next, we performed the GO enrichment analysis of the COVID-19-induced cluster. We found that the top 15 significantly enriched processes were associated with protein folding and stress response (Fig. 4b, Supplementary Table 5c). Terms such as response to unfolded proteins, cellular response to heat, and regulation of inclusion body assembly indicate activation of protective mechanisms against proteotoxic stress. Also, enrichment of chaperone cofactor-dependent protein refolding and “de novo” post-translational protein folding suggests an increased demand for molecular chaperones to maintain proteostasis. Finally, the enrichment of negative regulation of cellular component organization, protein-DNA complex disassembly, and regulation of protein modification by small protein conjugation or removal may reflect a cellular strategy to reprioritize resources toward protein quality control (Fig. 4b). To better understand the biological processes affected by differential expression, we analyzed upregulated and downregulated genes separately. Downregulated genes were only significantly enriched for the positive regulation of receptor-mediated endocytosis (Supplementary Fig. 8b, Supplementary Table 5c). This impaired endocytic trafficking might be a protective host response or a consequence of COVID-19 infection or stress.

Conversely, upregulated genes were strongly enriched in several stress response and protein quality control pathways. Key processes included responses to unfolded proteins, cellular response to heat, chaperone cofactor-dependent protein refolding and “de novo” post-translational protein folding, and regulation of RNA splicing and mRNA processing, highlighting activation of the heat shock response and proteostasis mechanisms (Supplementary Fig. 8b, Supplementary Table 5c). Additionally, the negative regulation of inclusion body assembly may indicate a protective role in preventing protein aggregation during SARS-CoV-2 infection.

### Analysis of DEGs in Sertoli cells

Our histological analysis revealed that acute COVID-19 induced DNA damage and apoptosis in Sertoli cells (Fig. 2a–f), prompting us to investigate how the disease affected the transcriptional landscape in this cell type. As mentioned above, the Sertoli cell cluster had the most unique DEGs (Fig. 3g). In total, this cluster displayed 205 DEGs, 105 downregulated and 100 upregulated. Some of the downregulated DEGs were JRKL-AS, MIR202HG, KITLG, and SCML1, and some of the upregulated ones were CMYA5, ZNF117, INSR, and ERV3–1. First, we validated these results by analyzing ERV3–1 levels using RNAscope, which were significantly upregulated in COVID-19 patients (Fig. 3h).

The GO enrichment analysis of all DEGs in Sertoli cells revealed that enriched biological processes were related to protein and neuron synthesis (Fig. 4c, Supplementary Table 5c). When analyzing DEGs by expression direction, downregulated genes were primarily associated with cytoplasmic translation and gene expression (Cytoplasmic Translation, Macromolecule Biosynthetic Process, Translation, Gene Expression, Ribosomal Small Subunit Assembly; Supplementary Fig. 8c, Supplementary Table 5c), suggesting a global reduction in protein synthesis machinery. Conversely, upregulated DEGs were enriched in processes such as negative regulation of cellular component organization, learning, and axon guidance (Supplementary Fig. 8c, Supplementary Table 5c). Taken together, these findings indicate a pattern of severe stress: translational shutdown combined with aberrant activation of neuronal-like guidance programs.

### Analysis of DEGs in Spermatogonia

Our analysis of histology sections from COVID-19 patients showed a significant drop in SSC numbers, an increase in DNA damage and apoptosis, and we wanted to understand how the disease affected the transcriptional landscape of the SSCs. We analyzed the spermatogonia cluster and found 331 DEGs in COVID-19 patients, 67 upregulated, such as NDUFAF3, FAU, TUBB4B, UBE2E3, and 264 downregulated (CNTNAP5, LRP1B, PLCG2, PRRC2C). We validated these results by analysing the expression of UBE2E3 and PRRC2C in spermatogonia by IF and RNAscope, respectively (Fig. 3h, Supplementary Figure 7b).

To gain more insights into the effects of COVID-19 on spermatogonia, we conducted a GO enrichment analysis of the DEGs. The analysis revealed that COVID-19 disrupts processes related to protein synthesis and gene expression, including cytoplasmic translation, macromolecule biosynthetic process, translation, and gene expression (Fig. 4d, Supplementary Table 5c). Moreover, processes such as biogenesis of ribosomes, ribosomal small subunits, and ribonucleoproteins, as well as the assembly of the ribosomal small subunit, rRNA processing, and rRNA metabolic processes. Terms related to the energy obtention in the electron transport chain (aerobic electron transport chain, mitochondrial ATP synthesis coupled electron transport, mitochondrial electron transport, cytochrome C to oxygen, and energy derivation by oxidation of organic compounds) were also enriched in spermatogonia DEGs. Put together, these suggest alterations in RNA metabolism, and energy obtention mechanisms.

When analysing DEGs by expression direction, downregulated genes were strongly enriched in processes related to cytoplasmic translation, macromolecule biosynthetic process, translation, gene expression, ribosome biogenesis/assembly, and the electron transport chain, indicating a global suppression of protein synthesis machinery and aerobic energy obtention (Supplementary Fig. 8d, Supplementary Table 5c). The net effect of this spermatogonial metabolic arrest (no formation of new ribosomes, reduced translation capacity, downregulated oxidative phosphorylation energy) could lead to the collapse of the spermatogonial stem cell pool we detected cytologically. In contrast, the upregulated DEGs did not show significant enrichment for any specific GO term (Supplementary Fig. 8d, Supplementary Table 5c).

### The SARS-CoV-2 viral signature is similar in the testes and the brain.

A common question is whether the phenotype observed in COVID-19 is driven by inflammation or by a SARS-CoV-2-specific signature. To address this question, we compared our snRNAseq data to the results already published by Yang et al. using human brain and choroid plexus. We used this database given that: a) DEGs analysis of the COVID-19 testes samples presented very high deregulation of genes related to nervous system, and to neurodevelopmental phenotypes (Fig. 4c); b) brain and testes share a lot of similarities at a genetic level, with both tissues sharing the expression of highest number of genes of any organs in the body^75^, and c) Yang et al. analyzed COVID-19 samples as well as Flu samples, so finding a SARS-CoV-2 signature that is independent of inflammation was granted. Using the method described earlier, we identified a list of DEGs that were common in the snRNAseq of COVID-19 testes and in the COVID-19 brain, specifically in the parenchyma and choroid plexus. (Fig. 4e, Supplementary Table 7a). We found 104 DEGs common in both tissues, 35 of those upregulated in testes and brain parenchyma (Fig. 4e, Supplementary Table 7a), and 21 downregulated in both tissues (Fig 4e, Supplementary Table 7a). Two genes were upregulated in SARS-CoV-2-infected tissues but downregulated in brain tissue with flu (LHFPL3 and ALK) (Supplementary Table 7a). Analyzing the choroid plexus, we found 16 genes upregulated in the testes and choroid plexus, and 5 downregulated in both tissues. We also found five DEGs that were upregulated in the two SARS-CoV-2-infected tissues but downregulated in the brain with flu (LRP1B, RBFOX1, OSMR-AS1, PRKAG2, and NALCN) (Fig. 4e, Supplementary Table 7a). We validated these results with IF and RNAscope. Consistent with our snRNAseq data, samples from COVID-19 patients showed higher signals for MYO5C and ST6GAL1 than controls. Meanwhile, AIDA and YWHAB signals were reduced in COVID-19 samples (Fig. 4f).

Comparative GO enrichment analysis of differentially expressed genes (DEGs) in the testes and parenchyma of COVID-19 patients revealed no statistically significant biological processes (Supplementary Fig. 9a, Supplementary Table 7b). When analyzing DEGs by expression direction, DEGs that were downregulated in both SARS-CoV-2-infected tissues were primarily associated with long-term synaptic potentiation, natural killer cell-mediated cytotoxicity, and negative regulation of G Protein-coupled receptor signaling pathway (Supplementary Fig. 10a, Supplementary Table 7b). These suggest that SARS-CoV-2 infection is linked to suppressed immune cytotoxicity from Natural Killer cells and altered neuromodulatory signaling across tissues. DEGs upregulated in both infected tissues did not reveal any significant biological process associated with these genes (Supplementary Fig. 10b, Supplementary Table 35). In contrast, the DEGs that were found to be upregulated in SARS-CoV-2 tissues but downregulated in flu revealed biological processes associated with neurotransmitter-gated ion channel clustering, postsynaptic membrane organization, regulation of synapse assembly, regulation of dendrite development, positive regulation of neuron projection development, regulation of neuron differentiation, sensory perception of sound and mechanical stimulus, which suggests that SARS-CoV-2 specifically induces neuronal-like signaling programs in non-neural tissues, which could indicate misexpression or repurposing of neuronal signaling genes, or altered cell-cell communication (Supplementary Fig. 10c, Supplementary Table 7b). Other significant terms related to tyrosine kinase signaling (peptidyl-tyrosine autophosphorylation and peptidyl-tyrosine phosphorylation) and metabolic regulation (negative regulation of lipid catabolic process, regulation of lipid catabolic process, negative regulation of lipid metabolic process, and negative regulation of catabolic process) suggest a metabolic reprogramming to support viral replication and promote cell survival. Lastly, the positive regulation of NF-κB transcription factor activity, together with the downregulation of natural killer cell-mediated cytotoxicity, indicates a decoupling of inflammation from effective antiviral killing, a hallmark of COVID-19 pathology. Put together, SARS-CoV-2 infection induces an aberrant activation of neuronal and synaptic signaling pathways, altered kinase and lipid metabolism, and inflammatory signaling uncoupled from effective immune cytotoxicity; a pattern not observed in influenza infection.

Next, a comparative GO enrichment analysis of DEGs between the testis and the choroid plexus of COVID-19 patients revealed significant enrichment of processes related to sodium ion transport, axon extension, and negative regulation of ligand-independent signal transduction and extrinsic apoptosis (Supplementary Fig. 9b, Supplementary Table 7b). The DEGs were also analyzed by expression direction. Directional analysis showed that DEGs downregulated in both SARS-CoV-2-infected tissues were mainly associated with vascular repair and regenerative processes, including VEGF and FGF signaling, endothelial cell chemotaxis, wound healing, DNA biosynthesis, and RNA processing (Supplementary Fig. 10d, Supplementary Table 7b), indicating a broad suppression of tissue repair, vascular remodeling, and proliferative capacity. In contrast, DEGs upregulated in both infected tissues were enriched for pathways involved in anti-apoptotic signaling, ion transport, axon extension, cell growth regulation, vascular and barrier transport, ERBB4 signaling, and receptor tyrosine kinase activation (Supplementary Fig. 10e, Supplementary Table 7b), consistent with a protected, survival-oriented state focused on barrier maintenance and controlled signaling. Finally, DEGs upregulated in SARS-CoV-2 but downregulated in influenza were predominantly associated with regulation of membrane potential, nutrient sensing, glucose and lipid metabolism, ATP production, nucleotide biosynthesis, energy reserve management, and GABAergic synaptic transmission (Supplementary Fig. 10f, Supplementary Table 7b). This SARS-CoV-2-specific signature reflects pronounced metabolic reprogramming that supports cellular survival under stress and viral replication, as well as neuronal-like signaling pathways potentially involved in immune modulation and barrier function. Together, these results indicate that SARS-CoV-2 induces a similar transcriptional program across testis and choroid plexus, characterized by suppressed vascular and regenerative pathways, enhanced anti-apoptotic and ion-transport signaling, and extensive metabolic remodeling favoring cellular survival and energy flexibility, distinguishing COVID-19 from influenza infection.

### COVID-19 phenotype persists after recovery.

Our results from fatal samples showed increased DNA damage and apoptosis in Sertoli cells and SSCs after acute SARS-CoV-2 infection. Thus, we asked whether this phenotype was conserved in patients who had recovered from COVID-19. To achieve this, we obtained eight testicular biopsies from recovered patients undergoing testicular sperm extraction (TESE) 1 to 12 months after the infection, and we compared them to 10 control TESE biopsies obtained from patients whom had a negative COVID-19 PCR done at the time of the biopsy, besides no history of SARS-CoV-2 infection in their clinical records (asymptomatic COVID-19 could not be ruled out since antibody determination was not performed in all patients) (Supplementary Table 8–9).

**Figure 5:**
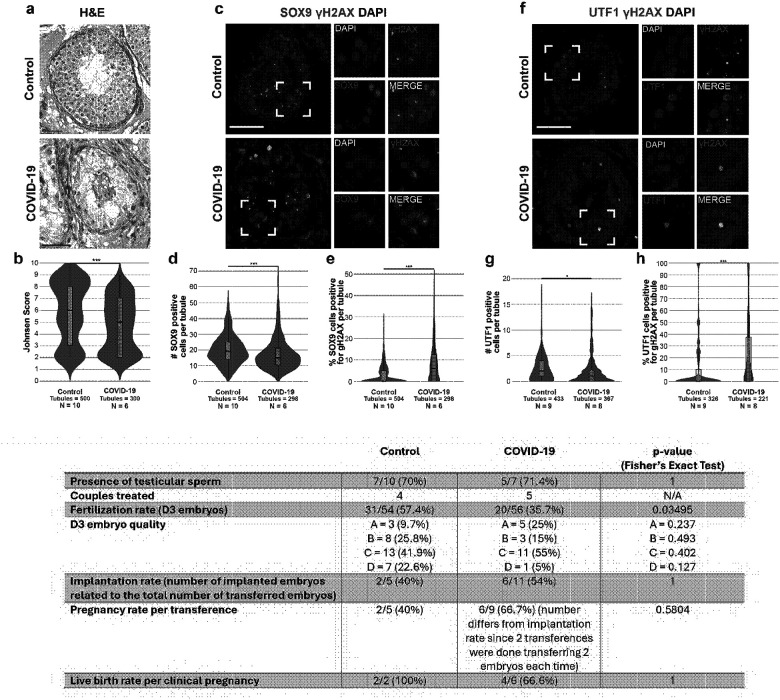
Legend not included with this version.

First, we sought to determine whether SARS-CoV-2 viral RNA could be detected in the testicular tissue of recovered patients. We were unable to detect viral RNA in seven testis biopsies from COVID-19 recovered patients (testing the eighth biopsy was not possible due to a lack of sample) (Supplementary Table 10). Next, we evaluated the general morphology of the testis using PAS-hematoxylin staining and scored the tubules following the Johnsen scoring system. We observed a tendency of a reduction in germ cells in COVID-19 biopsies, which corresponded with a lower Johnsen score (COVID-19 median = 5; control median = 6; Fig. 5a–b, Supplementary Table 11, p = 0.2547, Wilcoxon rank-sum test).

To analyze the long-term effects on Sertoli cells, we performed immunostaining with an SOX9 antibody. COVID-19 TESE biopsies showed a 21.1% loss of Sertoli cells (COVID-19 median= 15 cells per tubule, control median= 19 cells per tubule) (Fig. 5c–d, Supplementary Table 11, p= 0.0815, Student’s t-test). Since fatal samples from acute COVID-19 showed signs of DNA damage in Sertoli cells, we co-stained with gH2AX. 7.78% of the Sertoli cells of TESE COVID-19 were gH2AX+ compared to a 2.76% in controls (COVID-19 median = 1; control median = 0; Fig. 5c–e, Supplementary Table 11, p = 0.0013, Wilcoxon rank-sum test), indicating that Sertoli cells in recovered patients have increased DNA damage.

Permanent effects of SARS-CoV-2 on SSCs could have an essential impact on fertility^44,54^. Hence, to assess whether the reduction in SSCs observed during acute infection was maintained post-infection, we counted the number of undifferentiated SSCs using UTF1 staining. COVID-19 tubules had a median of 1 UTF1+ cell per tubule, compared to 2 in controls (Fig. 5f–g, Supplementary Table 11, p = 0.0398, Wilcoxon rank-sum test). Also, DNA damage was analyzed by double immunostaining for UTF1 and gH2AX. We found that COVID-19 samples had 99.45% more UTF1+gH2AX+ cells than the controls (Fig. 5f–h, Supplementary Table 11, p = 0.0303, Wilcoxon rank-sum test). Altogether, our results indicate that patients who recovered from COVID-19 have altered testicular morphology and reduced numbers of Sertoli cells and SSCs, as observed in fatal COVID-19 samples, which may affect fertility after infection.

### The COVID-19 persisted phenotype after recovery affects fertilization rate

To further assess this potential impact on male fertility, we analyzed clinical data from ICSI procedures performed with TESE samples from these individuals. Although testicular sperm were recovered from 7 controls and 5 COVID-19 recovered patients, only 4 controls and 5 COVID-19 recovered patients started ICSI with their respective partners. No female factor was documented. The fertilization rate of MII oocytes from the partners of COVID-19 recovered patients was significantly lower than that of the controls (COVID-19 = 35.7%, Control = 57.4%; p = 0.0349 Fisher’s Exact Test; Fig. 5 and Supplementary Table 12). The clinic’s reference fertilization rate for this period was 61%, confirming a decrease in control cases and a reduction of almost half of the fertilized oocytes in cases with a male partner who had recovered from COVID-19. Interestingly, the quality of the fertilized embryos at day 3 is similar between the two groups (Global: p = 0.162, A = 0.237, B = 0.493, C = 0.402, D = 0.127, Fisher’s Exact Test; Supplementary Table 25). Moreover, there is no significant difference between implantation rates (COVID-19 = 54%, Control = 40%; p = 1, Fisher’ Exact Test; Supplementary Table 12), pregnancy rate per transference (COVID-19 = 40%; Control = 66.7%; p = 0.5804, Fisher’ Exact Test; Supplementary Table 12), or live-birth rate per clinical pregnancy (COVID-19 = 66.6%, Control = 100%; p = 1, Fisher’ Exact Test; Supplementary Table 12), suggesting that the impact of COVID-19 affects mostly fertilization. It is important to note that the number of cases analyzed in this study is limited; therefore, while the trends observed are consistent with a potential effect of COVID-19 on reducing sperm fertilizing capacity, these findings should be interpreted with caution and require confirmation in larger cohorts.

## Discussion

Since the beginning of the COVID-19 pandemic, concerns have emerged regarding the potential effects of SARS-CoV-2 on male reproductive health. Previous studies have reported deleterious consequences of SARS-CoV-2 infection on testicular architecture, and our findings extend these observations by providing cellular and transcriptional insights into how COVID-19 impacts the human testis^50,51,76^.

Histological and cellular analyses revealed substantial disruption of testicular architecture in COVID-19 patients, including basement membrane detachment, cell sloughing into the tubular lumen, and a marked reduction in the intratubular cell population. These structural alterations were accompanied by apoptosis of testicular cells, as demonstrated by a significant increase in PARP1-positive cells and a trend toward increased TUNEL-positive cells, indicating enhanced apoptosis. The decrease in the intratubular cell population was closely associated with activation of the DNA damage response, as shown by increased gH2AX levels in COVID-19 samples but not in controls. In SSCs, elevated gH2AX correlated with significantly increased apoptosis, suggesting that DNA damage contributed directly to stem cell depletion. Notably, biopsies from patients who had recovered from COVID-19 showed the same pattern, with persistent loss of SSCs and Sertoli cells and sustained gH2AX expression, indicating that testicular damage may persist long after viral clearance.

SSCs are the most undifferentiated spermatogonia in the tissue and are essential for self-renewal, progression into differentiating spermatogonia, and then into spermatocytes that enter meiosis and ultimately give rise to spermatozoa^55,56^. Depletion of this population is a key contributor to infertility and reproductive aging. In line with our findings, a recent longitudinal study reported reduced sperm quality following mild COVID-19 infection, with total sperm count reduced by 57% in half of the patients compared to pre-infection levels^77^. In addition to stem cell loss, persistent DNA damage or impaired repair mechanisms may further compromise spermatocyte and sperm quality, increasing the risk of aneuploidy and miscarriage^78^.

Similar to SSCs, Sertoli cells also showed reduced cell numbers, increased apoptosis, and a trend toward elevated DNA damage. While DNA damage likely contributes to this phenotype, additional factors such as hypoxia and oxidative stress may also play a role. Given that Sertoli cells are essential for maintaining the SSC niche, forming the blood-testis barrier^63,79^, and regulating the biochemical environment required for spermatogenesis^57–59^, their dysfunction likely disrupts the testicular microenvironment, creating an imbalance in critical factors required for SSC maintenance, differentiation, and sperm formation. Consistent with these findings, we observed increased immune cell infiltration in COVID-19 testes, with marked increases in CD68-positive macrophages and CD3-positive T cells. Activated macrophages have been detected surrounding blood vessels and migrating into the testicular parenchyma in SARS-CoV-2 infection, where they may contribute to local inflammation and tissue damage^50,51^. The presence of T lymphocytes further suggests prolonged immune activation within the testis, which may amplify germ cell injury^50,51^.

At the transcriptional level, single-nucleus RNA sequencing revealed extensive remodeling of gene expression in COVID-19 testes. We identified 190 downregulated genes, including RABKDN, RPLP1, HIST1H2AA, and H2AFJ, which play key roles in meiosis, chromatin organization, and germ cell maintenance. RABKDN, a long noncoding RNA essential for meiotic progression, has been shown to regulate spermatogenesis and germ cell survival^80^, and its downregulation aligns with the histological defects observed here. Similarly, disruption of RPLP1 in animal models results in male infertility and impaired cell proliferation^81^. The downregulation of histone variants expressed in undifferentiated spermatogonia (HIST1H2AA and H2AFJ)^82,83^ further suggests altered chromatin regulation early in germ cell development. In contrast, several upregulated genes (PLCG2, LRP1B, RBFOX1, CDH13) are commonly associated with neurodevelopmental, neurodegenerative, and stress-related disorders. Although these genes have diverse functions, their coordinated upregulation suggests a broader transcriptional shift toward pathways typically linked to neural injury, immune activation, and cellular stress^84–89^. Together, these findings indicate that COVID-19 suppresses genes essential for germline integrity while activating pathways associated with cellular stress.

Interestingly, three genes involved in sperm binding to the zona pellucida (ADAM2^89,90^, LY6K^91,92^, and ZPBP2^93^) were downregulated in our global analysis. These genes are required for sperm-egg interaction and fertilization, and their reduced expression suggests impaired fertilization capacity in men who have recovered from COVID-19, a finding that is supported by our functional reproductive data. A COVID-19-induced cell cluster, comprising nearly 90% of COVID-19 derived cells, showed coordinated downregulation of genes involved in DNA packaging and histone replacement (TNP1^94^, PRM1^95^, HMGB4^96,97^), indicating impaired chromatin condensation during spermiogenesis. In parallel, this cell cluster showed upregulation of heat shock proteins involved in protein folding and stress responses (HSPA5^98,99^, HSPA1A^100,101^, HSPA1B^101^, STIP1^102^, DNAJB1^103,104^), consistent with increased proteostatic stress. Together, these transcriptional changes point to disrupted chromatin remodeling and increased cellular stress, both of which are critical for normal spermatogenesis and fertility. Consistently, sperm DNA condensation was downregulated in this cluster due to reduced expression of genes required for the histone-to-protamine transition, including TSSK6 and TNP^105,106^. Defects in this transition impair chromatin compaction and are strongly associated with male infertility^107^.

Cell-type-specific analyses further revealed that Sertoli cells downregulated genes required for germ cell support (JRKL-AS1, MIR202HG, KITLG, SCML1)^108,109^ while upregulating genes associated with neurological and immune-related disorders (CMYA5^110–112^, ZNF117^113,114^, INSR^115^, ERV3^116–118^). Similarly, spermatogonia showed downregulation of genes involved in proliferation and metabolism (NDUFAF3^119^, FAU^120,121^, TUBB4B^122,123^, UBE2E3^124^) and upregulation of genes linked to neurodevelopmental disorders and stress granule formation (CNTNAP5^125,126^, PLCG2^84,85,127^, LRP1B^87^, PRRC2C^128,129^). Across both cell populations, SARS-CoV-2 infection appears to trigger a coordinated cellular reprogramming marked by activation of membrane-trafficking, calcium-signaling, and neurite/axon-guidance pathways, alongside a broad suppression of protein-synthesis and metabolic machinery. Upregulated GO terms in both clusters converge on enhanced receptor-mediated endocytosis and tight regulation of calcium-mediated signaling, accompanied by the induction of multiple neuron-projection, axon-growth, and cytoskeletal-guidance programs, suggesting that infected cells remodel their membrane dynamics and intracellular transport systems in response to infection. At the same time, both cell populations show downregulation of ribosome biogenesis, rRNA processing, translation, protein metabolic processes, and other central biosynthetic pathways, reflecting a shared suppression of global protein synthesis capacity. This is further reinforced in spermatogonia by decreased mitochondrial electron transport and ATP synthesis, indicating metabolic dampening. Together, these results suggest that SARS-CoV-2 elicits a conserved response in both cell types characterized by increased trafficking and signaling plasticity paired with a deliberate shutdown of energetically costly biosynthetic and translational programs, a pattern consistent with a host antiviral state that reshapes cellular architecture while limiting resource availability for viral replication^130^.

Genes involved in epigenetic and chromatin regulation, cell cycle progression, and stress responses are highly disrupted in spermatogonia, so the long-term effects of the infection beyond 12 months need to be analyzed.

Pseudotime and trajectory inference analyses revealed altered differentiation dynamics in COVID-19 samples, with delayed progression and diversion into a secondary lineage, particularly during meiotic entry. These findings suggest impairments at the onset of meiosis and highlight a critical stage that needs further investigation. A key question is whether these effects reflect generalized inflammation or a SARS-CoV-2-specific transcriptional signature. Given the high dysregulation of genes involved in neurogenesis in the testis, we compared our data with published snRNA-seq datasets from SAR-CoV-2-infected and Influenza-infected brains. We identified shared transcriptional changes across testis, brain parenchyma, and choroid plexus that were specific to COVID-19 and distinct from those of Influenza. These findings support the existence of a SARS-CoV-2-specific signature rather than a nonspecific inflammatory response, since our data showed that at least two organs, the brain (parenchyma and choroid plexus) and testis, have a similar response, different from the Flu. It is important to note that our results were derived from unvaccinated populations during the acute phase (cadavers), so how this response differs across populations with different COVID-19 vaccine doses needs to be addressed.

Notably, the testicular features observed in fatal COVID-19 cases persisted for up to 12 months in recovered patients. Although this study is limited by sample size and the use of biopsies, our findings complement semen-based studies reporting transient alterations in sperm parameters following infections^77,131^. Unlike previous work focused on semen, our study provides direct evidence of persistent alterations in germ and somatic cells within the testis itself. Nonetheless, given the modest sample size, these results should be interpreted as preliminary and warrant continued investigation in larger, independent cohorts.

Finally, reproductive outcomes from recovered patients revealed a significant reduction in fertilization rates when using testicular sperm. This could be explained since sperm DNA fragmentation and seminal oxidative stress have been reported to be increased in patients with a history of COVID-19 infection, and injecting oocytes with suboptimal spermatozoa could translate into non-developing zygotes. However, it is important to acknowledge that the present observations were derived from cohorts of COVID-19–recovered men who already presented with fertility issues. Consequently, the alterations described here may not fully represent the general population. It remains possible that individuals with underlying subfertility are more vulnerable to post-infection testicular or spermatogenic impairments, and that the magnitude of the effects we observed may be amplified in this specific clinical subgroup. Future research will be essential to disentangle the contributions of pre-existing reproductive conditions from those directly attributable to SARS-CoV-2 infection. Larger, prospective, population-based studies, as well as mechanistic investigations, will clarify the long-term reproductive sequelae of COVID-19 and identify which male patients are most at risk.

In summary, our study demonstrates that SARS-CoV-2 infection is associated with sustained structural, cellular, and transcriptional alterations in the human testis. COVID-19 leads to the depletion of SSC and Sertoli cells, persistent DNA damage, increased apoptosis, immune cell infiltration, and profound disruption of chromatin remodeling and stress-response pathways. These changes converge on key processes required for germ cell maintenance, meiotic progression, and sperm function, and are accompanied by transcriptional signatures shared with SARS-CoV-2–infected neural tissues. Importantly, these alterations persist in recovered patients and are associated with reduced fertilization capacity, suggesting that SARS-CoV-2 infection may have lasting consequences for male reproductive health. While further longitudinal and mechanistic studies are needed, our findings provide direct evidence that SARS-CoV-2 infection can compromise testicular homeostasis and male fertility beyond the acute phase of disease.

## Materials and methods

### Testicular samples

Samples were obtained from the Office of the Chief Medical Examiner (OCME) in Oklahoma City, OK, USA, where autopsies followed guidelines from the National Association of Medical Examiners (NAME) and the College of American Pathologists (CAP). Additional samples came from the Center for Organ Recovery and Education (CORE) with approval from the University of Pittsburgh Committee for Oversight of Research and Clinical Training Involving Decedents (CORID protocol #686, titled Fertility Preservation Program; PI Kyle Orwig), from Mater Dei Hospital in Belo Horizonte, Brazil (approved by the National Research Ethics Committee (CONEP) under number CAAE: 30999320.1.0000.5128), from Hospital Clínic (IDIBAPS Biobank, Ethics Committee project A2-C21027) and Hospital de Bellvitge in Barcelona, Spain (Ethics Committee project BB20–024), from Biobanco Hospital Universitario Puerta de Hierro Majadahonda (HUPHM, Ethics Committee project 03BTP21), Madrid, Spain, and from Biobanco Vasco (Ethics Committee project TX200), Basque Country, Spain. All samples were collected and handled according to the ethical guidelines of each respective center or biobank. All patients enrolled, aged from 25 to 88 years old, had deceased from COVID-19 complications, with diagnosis confirmed by SARS-CoV-2 RT-qPCR during their hospital stay. Control samples were obtained from the Center for Organ Recovery and Education (CORE) with approval from the University of Pittsburgh Committee for Oversight of Research and Clinical Training Involving Decedents (CORID protocol #686, titled Fertility Preservation Program; PI Kyle Orwig). These controls were age-matched and collected before the pandemic. Control samples from Spain were obtained from Hospital Clínic (IDIBAPS Biobank) and Hospital de Bellvitge, Barcelona, Spain; and from Biobanco Hospital Universitario Puerta de Hierro Majadahonda (HUPHM), Madrid, Spain.

For the histological analysis, samples of testes of different origin and age were obtained from unvaccinated patients who died of COVID-19 in the U.S, Spain, and Brazil (n= 28, Supplementary Table 1). Samples of testes from males who died in the U.S. and Spain by causes other than an infectious disease were collected as controls (n= 23, Supplementary Table 1). Tissues were obtained postmortem and fixed in 4% paraformaldehyde, in 10% neutral-buffered formalin, or in Bouin’s fixative (24% formalin, 5% glacial acetic acid in picric acid). For the snRNAseq analysis, testis fragments were sampled and then snap-frozen in liquid nitrogen. Samples of testes of different age were obtained from patients who died of COVID-19 in Brazil (n=5), and those were compared with same age samples obtained from control testes, from males who died in the U.S. by causes other than an infectious disease (n= 5, Supplementary Table 1). Supplementary Table 13 shows which samples were used for each experiment.

### Testicular Sperm Extraction (TESE)

TESE samples were obtained from Fundació Puigvert and Clinica Gravida in Barcelona, Spain. Ten control samples and eight COVID-19 recovered samples were collected via a median raphe incision under local or general anesthesia (Supplementary Table 8). Testicular blood vessels within the tunica albuginea were identified with 8–15x optical magnification. Most of the recovered tissue was used for IVF/ICSI purposes; 10% was fixed in Bouin’s fixative (24% formalin, 5% glacial acetic acid in picric acid), and 10% was snap-frozen in liquid nitrogen and stored at −80°C for research. Of these samples, only seven controls and five COVID-19 samples had testicular spermatozoa, making ICSI possible. The rest had severe lesions and abandoned their reproductive efforts, except for one couple from the control group who did FIV/ICSI with donor semen. Among the seven control patients with positive biopsies, one had no partner and two are not on an IVF waitlist, so they also abandoned their reproductive plans. Of the nine patients who underwent ICSI, there were 8 pregnancies—two with partners from control patients and six from COVID-19 patients. Both pregnancies in control partners resulted in births, while two of the six in COVID-19 partners ended in abortions. Three additional embryo transfers per group were performed but did not result in pregnancy. Three couples could not conceive, but one still has up to six frozen embryos after PGT-SR that could be transferred (Supplementary Table 9).

### Sample transport and histological processing.

Testis used for histology and immunofluorescence were dissected, fixed with 10% buffered formalin for 21 days, and then transferred to 70% ethanol. They were shipped to the laboratory, washed three times in 70% ethanol, embedded in paraffin, and sectioned at 5 μm for use in periodic acid-Schiff (PAS) - Hematoxylin staining, immunohistochemistry, and RNAscope. Frozen testicular samples were shipped from Brazil to U. Pitt on dry ice and stored at −80°C until used for single nuclei RNA-seq.

### PAS-Hematoxylin staining

PAS-Hematoxylin staining was performed on paraffin-embedded sections. Slides were deparaffinized and rehydrated with 3 washes of Xylene for 10 minutes each, followed by 3 washes of each concentration in a graded series of ethanol (100%, 95%, 80%, 70%) for 5 minutes. After deparaffinization and rehydration, slides were oxidized for 10 minutes with 1% Periodic Acid Schiff, washed with water, stained with Schiff’s reagent for 10 minutes in darkness, washed with sulphureous water, and counterstained with hematoxylin. The sections then were dehydrated and mounted in Cytoseal^™^60 or DPX. A visual assessment of the stained samples was performed to detect the differences between COVID-19 and control tissues.

### Johnsen Score assessing

The quality of the samples was assessed through the Johnsen scoring system, in which spermatogenesis in the seminiferous tubules is evaluated. 50 tubule cross-sections were scored per sample, with each one given a score from 10 to 1, with 10 being a tubule with full spermatogenesis, and 1 a tubule with no seminiferous epithelium. The intermediate scores are as follows: 9: slightly impaired spermatogenesis, many late spermatids; 8: less than 5 sperm per tubule, few late spermatids; 7: no sperm, no late spermatids, many early spermatids; 6: no sperm, no late spermatids, few early spermatids; 5: no sperm or spermatids, many spermatocytes; 4: no sperm or spermatids, few spermatocytes; 3: spermatogonia only; 2: no germinal cells, Sertoli cells only^60^.

### Immunohistochemistry on paraffin-embedded samples

Samples were deparaffinized and rehydrated, then submerged 40 minutes in sodium citrate at 100°C for antigen retrieval, cooled down for 20 minutes, rinsed in PBS-Tween-20, permeabilized with PBS-Triton X-100 for 30 minutes, rinsed in PBS-Tween-20, blocked with 10 % blocking solution in PBS (1% 10x PBS, 1% goat serum, 0.3% BSA, 0.025% glycine, 0.02% Triton X-100 and 0.01% Tween-20) for 4 hours, and incubated in the primary antibody overnight at room temperature (Supplementary Table 14). For the CD68 immunostaining, DAKO Antibody Diluent, Background Reducing (Agilent) was used to incubate the slides for 15 minutes before and after the blocking and to dilute the antibodies. Anti-CD3 and CD68 antibodies were used to detect immune infiltrates in the peritubular tissue of the testis; anti-cleaved PARP1 was used to detect cell death; anti-SOX9 was used to mark Sertoli cells in the tubules; anti-UTF1 was used to detect undifferentiated spermatogonial stem cells in the tubules; and gH2AX was used to detect DNA damage. The following day, the sections were rinsed in PBS-Tween-20, incubated with the secondary antibody (Supplementary Table 15) for 1 to 2 hours at room temperature, rinsed in PBS-Tween-20, counterstained with DAPI to visualize the nuclei, and mounted in VECTASHIELD^®^. Image acquisition was performed using a Zeiss AxioImager M2 microscope (Carl Zeiss AG, Oberkochen. Germany). Images were processed using Zen 2 (Carl Zeiss AG, Oberkochen. Germany).

### TUNEL assay

To detect apoptotic cells, the Click-iT^™^ Plus TUNEL Assay Kit (Invitrogen, C10617) was used. For sections with immunohistochemistry for SOX9 or UTF1, the slides were submerged in 4% PFA for 15 minutes at 37°C, rinsed with PBS, covered in proteinase K solution for 15 minutes, rinsed again with PBS, then immersed in 4% PFA for 5 minutes at 37°C, and rinsed in PBS and deionized water. Next, the TdT reaction was performed by incubating the slides for 1 hour at 37 °C, followed by the Click-iT Plus reaction with Alexa Fluor^®^ 488 for 30 minutes at 37 °C. The sections were then counterstained with DAPI and mounted with VECTASHIELD^®^. Image acquisition was carried out using a Zeiss AxioImager M2 microscope (Carl Zeiss AG, Oberkochen, Germany). Images were processed using Zen 2 (Carl Zeiss AG, Oberkochen, Germany).

### RNAscope assay

RNA detection was conducted using the RNAscope Fluorescent Multiplex kit (catalog no. 323135; Advanced Cell Diagnostics, Newark, CA) following the manufacturer’s instructions with slight modifications. Briefly, target probes (Supplementary Table 16) were obtained from Advanced Cell Diagnostics. Paraffin-embedded sections were deparaffinized three times with xylene, each for 5 minutes, then washed twice with 100% ethanol for 2 minutes each. Next, tissues were treated with hydrogen peroxide for 10 minutes, heated in the kit-provided target retrieval buffer for 15 minutes at 100°C, and digested with the provided proteinase plus. After digestion, probes were added and incubated at 40°C for 2 hours. The probes were then rinsed, and the signal was amplified using the preamplifier reagent, followed by the addition of Opal fluorophores. The reaction was blocked, sections were counterstained with DAPI, and mounted with VECTASHIELD^®^. Image acquisition was performed using a Zeiss AxioImager M2 microscope (Carl Zeiss AG, Oberkochen, Germany). Images were processed with Zen 2 (Carl Zeiss AG, Oberkochen, Germany).

### Cell counting

For the CD3 and CD68 counting, 50 peritubular spaces were analyzed, counting the total DAPI positive cells and the CD3 or CD68 positive cells. The results shown are the proportion of CD3 or CD68 positive cells versus all the cells present in that peritubular space. This counting was performed under the fluorescence microscope. For the rest of the counting (PARP1+, SOX9+, SOX9+gH2AX+, SOX9+TUNEL+, UTF1+, UTF1+gH2AX and UTF1+TUNEL+), tile pictures at 40x magnification were taken from sections of the testes, and then counted using QuPath (version 0.3.2). Around 150 tubules per sample were selected, counting all the DAPI positive cells, and the cells with specific signal (PARP1, SOX9 or UTF1). For gH2AX signal, only the ones colocalizing with SOX9 or UTF1 were counted. For TUNEL signal, first, all positive cells were counted, and then the ones colocalizing with SOX9 or UTF1 signals were counted. The results are shown as a total number (for DAPI, SOX9 and UTF1), as a proportion of SOX9/UTF1 positive cells per tubule; and as a proportion of SOX9/UTF1 cells gH2AX/TUNEL positive cells per tubule.

### RNA extraction from paraffin-embedded sections

RNA was extracted from paraffin-embedded sections using the RNeasy FFPE Kit from Qiagen according to the manufacturer’s instructions. Two sections per sample were scraped with a scalpel, placed in 1 ml of xylene, and centrifuged at full speed for 2 minutes. The supernatant was discarded, and 100% ethanol was added to the pellet, vortexed, and centrifuged again at full speed for 2 minutes. After discarding the supernatant, the tube was left open to dry. Buffer PKD and proteinase K were added and incubated at 56°C for 15 minutes, then at 80°C for 15 minutes, and cooled on ice for 3 minutes. The mixture was then centrifuged for 15 minutes at 13,500 rpm, and the supernatant transferred to a new microcentrifuge tube, followed by adding DNase Booster Buffer and DNase I stock solution, and incubating for 15 minutes. Next, Buffer RBX was added, and the lysate was mixed thoroughly. 100% ethanol was added, mixed by pipetting, and immediately transferred to an RNeasy MinElute spin column placed inside a 2 mL collection tube, then centrifuged for 30 seconds at 12,000 rpm. The flowthrough was discarded, and this step was repeated until the entire sample had passed through the column. Buffer RPE was added to the column, which was then centrifuged for 30 seconds at 12,000 rpm. The flowthrough was discarded, and Buffer RPE was added again, followed by centrifugation for 2 minutes at 12,000 rpm. The collection tube containing the flowthrough was discarded, and the spin column was placed in a new collection tube. Then, 15 μL of RNase-free water was added directly onto the column membrane, and centrifuged for 1 minute at full speed to elute the RNA. This step was repeated to achieve a final volume of 30 μL. The concentration of RNA was determined using a NanoDrop spectrophotometer, and the samples were stored frozen at −80°C.

### RT-PCR

To test for the presence of the virus in the testes, we performed a quantitative real-time PCR (qRT-PCR) using a commercial kit that detects two regions of the SARS-CoV-2 nucleocapsid (N1 and N2). We were able to detect the virus in 6 of our testes tissue samples but not in the TESE samples. The Bio-Rad Reliance SARS-CoV-2 RT-PCR IVD Assay Kit was used for the qualitative Real-time RT- PCR to detect two regions of the SARS-CoV-2 nucleocapsid gene (N1 and N2), and a constitutively expressed human RNase P gene. The sample RNA was thawed, and the master mix was prepared following the manufacturer instructions. Briefly, 10 μL of the master mix were dispensed in the number of wells needed of an RT-PCR plate (5 μL Reliance one-step multiplex RT-qPCR supermix, 1.5 μL SARS-CoV-2 RT-PCR Oligos, 3.5 μL nuclease free water). Ten microliters of RNase/DNase free water were placed in one well for an NTC, 10 μL of negative control material was placed in a Negative Control well and 10 μL of positive control material was added to a Positive Control well. In the remaining wells, 10 μL of extracted RNA sample was placed in each well. The plate was sealed with sealing film and centrifuged for 30 seconds at 1000 RCF. Then, the plate was loaded onto a CFX96 machine (Bio-Rad), to perform the RT-qPCR protocol (Supplementary Table 17). The fluorophores used in this assay were FAM for N1 protein from SARS-CoV-2, HEX for N2 protein from SARS-CoV-2 and Texas, for RNase P, which served as a control.

### RNA extraction from snap frozen tissue

The RNA extraction for its later use in ddPCR was done with TRIzol Reagent, which was added to the tissue and then homogenized in the homogenizer. After a 5 minute incubation to allow for the complete dissociation of the nucleoprotein complex, chloroform was added and thoroughly mixed to lysate the tissue. Then, samples were centrifuged for 15 minutes at 12000g at 4°C, which allows the mixture to separate into a lower phenol-chloroform phase, an interphase and a colorless upper aqueous phase. This aqueous phase was transferred to a new tube and RNAse-free glycoblue was added, which gave the RNA a blue color, in order to identify the pellet later. Isopropanol was then added to the aqueous phase and incubated overnight at −20°C. The next day, samples were centrifuged for 10 minutes at 12000g at 4°C, and the RNA precipitate forms a gel-like pellet at the bottom of the tube. The supernatant was discared and the pellet resuspended in 75% ethanol, and centrifuged for 5 minutes at 7500g at 4°C. Once again, the supernatant was discarded and the RNA pellet air dried for 5–10 minutes, before resuspending it with 25 μL of RNAse-free water. RNA was stored at −8’°C.

### Isolation of nuclei from frozen testes

The nuclei were isolated using the Nuclei PURE Prep Nuclei isolation kit (Millipore Sigma). Frozen testes were thawed, placed in PBS 1x, minced with a scalpel, and incubated in lysis buffer for 1 hour at 4°C on a shaker. They were then centrifuged for 5 minutes at 500 rpm at 4°C, with the supernatant discarded. The pellet was resuspended in cold PBS 1x, filtered through a 40 μm cell strainer, centrifuged again, and after removing the supernatant, finally resuspended in Nuclei PURE storage buffer.

### Generation of libraries for snRNAseq

The libraries for the droplet-based snRNAseq were generated using the Chromium Next GEM Single Cell 3 v.3.1 according to the manufacturer’s protocol. The cells were counted with a hemocytometer, targeting 10,000 nuclei per sample. Fifteen cycles were applied to generate cDNA, all samples underwent 15 or 16 cycles for final library generation. Generated snRNA-seq libraries were sequenced on a NovaSeq 6000 (NovaSeq 6000 S2 Reagent Kit v1.5 (100 cycles, Novogene).

### snRNAseq data analysis, QC, cell annotation, and DEG analysis

Raw sequencing reads were pre-processed with CellRanger v6.1.2 (10X Genomics) using the human reference genome (GRCh38–2020-A, 10X Genomics) to generate a digital expression gene-cell UMI matrix. The barcodes corresponding to background noise were removed during pre-processing. scDblFinder v1.10.0 was applied to exclude potential doublets^132^. Scater v 1.24.0 was used to identify outliers based on library size and the number of expressed genes^133^. The outliers were determined individually for each dataset. Cells with a value higher or lower than 3x median absolute deviation (MADs) of the median value for each metric were removed. Seurat v4.3.0 in R v4.2.0 was used for the downstream^134^ Cells in which <5% of reads were from mitochondria were kept. Genes detected in at least 3 cells were retained. The data were normalized using “sctransform” function in Seurat. The cell cycle state of each cell was determined based on their expression of G2M and S phase genes. The noise from UMI variation was regressed out of the data during data scaling. *Principal component analysis* (PCA) was done after identifying highly variable genes. The batch effect was corrected with Harmony v0.1.0^135–138^. The significant principal components were used for clustering cells and as input to uniform manifold approximation and projection (UMAP) for dimensionality reduction to facilitate visualization in 2 dimensions. Cell clusters and marker genes for each cluster were determined with Seurat. The cell-type identity for each cluster was scored by ScType (https://github.com/IanevskiAleksandr/sc-type)^139^, then refined by manual expert review of the cluster marker genes. Differential gene expression of genes comparing the COVID-19 and control groups was done using Seurat v4.3.0 with the MAST algorithm^135^. Thresholds of log-transformed fold change >= 2 (absolute value), adjusted *P* value (Bonferroni correction) < 0.05, and expression in greater than 10% of cells were required to consider a gene differentially expressed. The gene ontology enrichment analysis was performed using enrichR v3.1 with all parameters kept as default^136,137^.

### Trajectory analysis

Based on the cell type annotation, germ cells were selected for modeling developmental trajectories. Trajectories were inferred using slingshot v2.4.0^72^, with UMAP embeddings provided as the input dimensionality reduction. Following trajectory inference, trajectory-based differential analysis between experimental groups (Covid vs Control) were conducted using condiments v1.4.0^71^. To assess the presence of a shared trajectory structure across conditions, the topologyTest function was applied. As the test rejected the null hypothesis of a common trajectory, one trajectory per condition was refitted using the slingshot_conditions function. Differences in differentiation progression along the trajectories were evaluated using progressionTest().

### Statistics

All the statistical analysis were performed with R, using the following packages: car, dplyr, ggplot2, grid, gridExtra, here, RColorBrewer, readxl, tibble, tidyr, writexl. The significant p-value was determined as p<0.05. No statistical methods were used to predetermine sample size. The experiments were not randomized, and investigators were blinded to allocation during experiments and outcome assessment. For the histological countings, the mean, median and standard deviation were calculated for each sample in each experiment. The statistical test followed was chosen after performing a normality test and a homogeneity of variances test. A Shapiro-Wilk normality test was performed, with a p-value < 0.05 meaning that the samples did not follow a normal distribution. Next, a Levene’s test was performed to test for the homogeneity of the variances in the samples, if p-value < 0.05, the variances were not homogeneous. If the samples followed normality, the following statistical tests were done using the means, but if they did not follow normality, the statistical tests were done using the median. If the samples followed normality and had homogenous variances, a Student’s t-test was performed. If the samples followed normality but did not have homogenous variances, Welch’s test was performed. Finally, if the samples did not follow normality, with or without homogeneous variances, a Wilcoxon rank-sum test was performed. A Grubbs’ test was performed in Graphpad (https://www.graphpad.com/quickcalcs/grubbs1/) to detect outliers in each cohort for each experiment.

If outliers were detected, the values were excluded and the normality test, homogeneity of variances and the statistical tests were performed again, to confirm that the statistical results were not due to these outliers.

## Supplementary Material

This is a list of supplementary files associated with this preprint. Click to download.

- Fig.Supp3new.pdf
- SupplementaryTable17.qRT.docx
- SupplementaryTable12.Summaryofreproductivedataversion2.docx
- Supplementarytable10.RTqPCRTESE.xlsx
- SupplementaryTable11.Characterizationofrecoveredpatients.docx
- Supplementarytable9.ReproductiveinformationofTESEsamples.docx
- SupplementaryTable6.DEmarkersdifferentialglobalControlvsCovidallcells.xlsx
- Supplementarytable2RTPCR.docx
- SupplementaryTable13.SamplesusedineachexperiementVERSION2.docx
- Fig.Supp92.pdf
- SupplementaryTable1.Samplesummarymainanalysisnew.docx
- SupplementaryTable14.Primaryantibodies.docx
- Fig.Supp8.pdf
- SupplementaryTable4.snRNAseqmarkers.xlsx
- Fig.Supp7new.pdf
- Supplementarytable3Characterizationoffataltestistissue.docx
- Fig.Supp5new.pdf
- Fig.Supp4new.pdf
- SupplementaryTable16.RNAscopeprobes.docx
- SupplementaryTable5.MarkersdifferentialControlvsCovid.xlsx
- SupplementaryTable7.TestisvsBrain.xlsx
- SupplementaryTable8.SummaryofTESEsamples.docx
- Fig.Supp2new.pdf
- Fig.Supp102.pdf
- SupplementaryTable15.Secondaryantibodies.docx
- Fig.Supp6new.pdf
- Fig.Supp1new.pdf

## Data Availability

The snRNAseq data sets generated for this study are deposited in the NCBI GEO repository, under the accession number GSE324409. The brain RNAseq data re-analyzed for this study can be found at GSE159812. Source data are provided with this paper.
